# The DendrisCHIP^®^ Technology as a New, Rapid and Reliable Molecular Method for the Diagnosis of Osteoarticular Infections

**DOI:** 10.3390/diagnostics12061353

**Published:** 2022-05-30

**Authors:** Elodie Bernard, Thomas Peyret, Mathilde Plinet, Yohan Contie, Thomas Cazaudarré, Yannick Rouquet, Matthieu Bernier, Stéphanie Pesant, Richard Fabre, Aurore Anton, Cathy Maugis-Rabusseau, Jean Marie François

**Affiliations:** 1 Dendris SAS, 335 rue du Chêne Vert, F-31670 Labège, France; ebernard@dendris.fr (E.B.); tpeyret@dendris.fr (T.P.); mplinet@dendris.fr (M.P.); ycontie@dendris.fr (Y.C.); tcazaudarre@dendris.fr (T.C.); spesant@dendris.fr (S.P.); aanton@dendris.fr (A.A.); 2 Laboratoire CBM-Inovie, F-31000 Toulouse, France; yannick.rouquet@inovie.fr (Y.R.); matthieu.bernier@inovie.fr (M.B.); richard.fabre@inovie.fr (R.F.); 3 Institut de Mathématiques de Toulouse, UMR5219, Université de Toulouse, CNRS, INSA, F-31077 Toulouse, France; cathy.maugis@insa-toulouse.fr; 4 TBI, Université de Toulouse, CNRS, INRAE, INSA, 135 Avenue de Rangueil, F-31077 Toulouse, France

**Keywords:** bone and joint infection, in vitro multiplex diagnostic, biochips, next-generation sequencing, microbial cultures

## Abstract

Osteoarticular infections are major disabling diseases that can occur after orthopedic implant surgery in patients. The management of these infections is very complex and painful, requiring surgical intervention in combination with long-term antibiotic treatment. Therefore, early and accurate diagnosis of the causal pathogens is essential before formulating chemotherapeutic regimens. Although culture-based microbiology remains the most common diagnosis of osteoarticular infections, its regular failure to identify the causative pathogen as well as its long-term modus operandi motivates the development of rapid, accurate, and sufficiently comprehensive bacterial species-specific diagnostics that must be easy to use by routine clinical laboratories. Based on these criteria, we reported on the feasibility of our DendrisCHIP^®^ technology using DendrisCHIP^®^OA as an innovative molecular diagnostic method to diagnose pathogen bacteria implicated in osteoarticular infections. This technology is based on the principle of microarrays in which the hybridization signals between oligoprobes and complementary labeled DNA fragments from isolates queries a database of hybridization signatures corresponding to a list of pre-established bacteria implicated in osteoarticular infections by a decision algorithm based on machine learning methods. In this way, this technology combines the advantages of a PCR-based method and next-generation sequencing (NGS) while reducing the limitations and constraints of the two latter technologies. On the one hand, DendrisCHIP^®^OA is more comprehensive than multiplex PCR tests as it is able to detect many more germs on a single sample. On the other hand, this method is not affected by the large number of nonclinically relevant bacteria or false positives that characterize NGS, as our DendrisCHIP^®^OA has been designed to date to target only a subset of 20 bacteria potentially responsible for osteoarticular infections. DendrisCHIP^®^OA has been compared with microbial culture on more than 300 isolates and a 40% discrepancy between the two methods was found, which could be due in part but not solely to the absence or poor identification of germs detected by microbial culture. We also demonstrated the reliability of our technology in correctly identifying bacteria in isolates by showing a convergence (i.e., same bacteria identified) with NGS superior to 55% while this convergence was only 32% between NGS and microbial culture data. Finally, we showed that our technology can provide a diagnostic result in less than one day (technically, 5 h), which is comparatively faster and less labor intensive than microbial cultures and NGS.

## 1. Introduction

Orthopedic prosthetic joint infection (PJI) is a specific osteoarticular infection related to joint arthroplasty, with potential dreadful complications that may require long and expensive treatment [1,2]. With the steady rise in the aging population, prosthetic surgery is going to increase dramatically, mostly in industrialized countries. For instance, a projected increase of 2 to 5 fold for primary hips and knees arthroplasties has been estimated in the United States between 2005 and 2030 [3]. Consequently, a parallel increase in PJI is expected, with a projected prevalence exceeding 60,000 to 70,000 patients in the United States by 2020 [4]. In France, the prevalence is approximately 70 infections per 100,000 primary arthroplasties, the overall rehospitalization rate is about 18.3%, with a disability rate of 40%, and an amount of fatality cases of around 5% [5,6]. For these reasons, a prompt and decisive identification of the causative organism(s) is critical in the management of patients with PJI as inferred by the European Bone and Joint Infection Society (EBJIS) [7]. However, this task remains very difficult due to the lack of rapid, reliable, standardized, and cost-effective diagnosis methods. An additional problem is the amount of pitfalls and errors that can occur during the process of diagnosing PJI, which can lead to a misdiagnosis and a mismanagement of infections [8].

While conventional microbiological culture-based methods deserve a central place in the recommendations concerning the etiological diagnosis and antibiotic treatment of PJI [9], these techniques have serious limitations as they lead to false-negative (cultures negative) results in up to 50% of PJI cases (reviewed in [10]). False-negative cultures notably pose serious problems in PJI diagnosis as they are responsible for about 4.5 times increased risk of reinfection in comparison with culture-positive cases. These negative cultures can be attributed to several reasons including previous antibiotic treatment, bacterial adherence to abiotic material leading to biofilms formation, insufficient culture time, inappropriate culture conditions, etc. [4,11]. In addition, the microbial culture technique is relatively dependent on the expertise of the laboratory technician who decides whether or not to pick the right colonies for analysis. As Esteban et al. [12] pointed out in a recent review, conventional microbiological cultures have probably reached their maximum efficiency. They advocated that the future of PJI diagnosis, as well as other areas of clinical microbiology and virology diagnostic, lies in molecular biology techniques, as they rely on genomic fingerprints that are the identity card of every biological species. These techniques should ideally be able to detect any microbial agent causing an infection with high sensitivity and specificity. So far, the most used molecular biology techniques are broad-range PCR on the *16S rRNA* gene [13,14,15], multiplex PCR using a commercially available device such as the Unyvero i60 ITI^®^ system from Curetis AG [16,17,18,19], or the FilmArray^®^ from BioFire Diagnostic [20]. One limitation of these PCR methods is their restriction to identifying the limited number of causative agents for which primers have been designed. To overcome this limitation, the next-generation sequencing (NGS) targeted on the *16S rRNA* gene sequencing or by shotgun metagenomics is an alternative molecular method currently evaluated in several clinical laboratories [11,21,22,23]. The power of these sequencing methods may also be their Achilles heel because they will give a complete picture of the microbial profile present in the sample, which may be irrelevant because of the absence of any symptomatic sign of infection [11,23]. Nonetheless, comparative studies have not yet determined whether these molecular methods could ever replace microbiological cultures, as, in most of these reports, the performance of these molecular methods in terms of sensitivity and specificity was not significantly superior to that of microbial cultures. However, there is a consensus that these molecular methods will be of great help in the diagnosis of PJI, which are (i) a rapid result in less than 24 h, which cannot be obtained by microbial cultures [12]; (ii) a high positive predictive value already obtained from a single specimen analyzed whereas at least three independent microbial cultures are recommended to reach a similar predictive value; and (iii) a diagnosis that can be performed on prosthetic tissues, for which microbial culture is less sensitive and specific while this type of sample is more readily available than synovial fluids or sonic fluid [14,21]. In addition to these technical aspects, the molecular approach to in vitro diagnostics raises the question of the need for standard references that characterize a healthy individual. This necessity is even more acute in the case of NGS on shotgun metagenomics, which has the power to decipher the microbiome in any type of human tissue or organ [24]. In addition to these issues, a new diagnostic method must meet several practical requirements, including ease of routine use in clinical laboratories, semiautomation, delivery of results in less than one day and, not least, a very favorable cost effectiveness. This highlights the need for a standardized test and protocol for the diagnosis of PJI, and in particular, to define the gold standard of agents actually responsible for this disease.

In this perspective, the aim of this work was to evaluate the feasibility of our DendrisCHIP**^®^** technology presented in a previous report [25] for the detection and identification of bacteria potentially implicated in osteoarticular infections. This technology is based on the principle of microarrays [26,27,28,29] in which the hybridization signals between oligoprobes attached to our proprietary DendriSLIDE^®^ [30,31] and targeted complementary labeled nucleic acid queries a database of hybridization signatures corresponding to a list of pre-established bacteria by a decision algorithm based on machine learning methods. This decision algorithm allows, on the one hand, to give a probability of presence or absence of bacteria in the sample and, on the other hand, to identify the bacteria at the genus or species level. Diagnostic results by the DendrisCHIP**^®^** technology can be obtained in less than 5 h, which is thus comparable to most multiple PCR technologies and by far faster than microbial cultures. In addition, compared to our previous report, we further developed a semiautomated sample processing from PCR purification to the reading of the DendrisCHIP**^®^**, which reduces the risks of contaminations and standardizes the process. This paper focused on the validation of the DendrisCHIP**^®^**OA which carries oligoprobes to specifically detect the principal bacteria involved in PJI, by performing a qualitative and quantitative comparison with microbiological cultures and NGS, with no intention at this stage to relate any of the obtained results with patient classification and clinical data and their interpretation. Overall, our results showed the high robustness of DendrisCHIP**^®^** technology that quantitatively compared with NGS technology whereas the concordance of detection was in the range of 60% with microbial cultures.

## 2. Materials and Methods

### 2.1. Bacterial, Clinical Specimens, and DNA

#### 2.1.1. Bacterial and Clinical Specimens Provision

The pure bacterial strains listed in Table 1 were kindly provided by the clinical laboratories of Toulouse or purchased from Orgentec SASU (Trappes, France). A total of 315 samples were collected from patients during surgery and provided by independent clinical laboratories of Toulouse as articular synovial fluid, deep-tissues specimens, or swabs. These samples were simultaneously analyzed by conventional bacteriological cultures by these laboratories according to their internal practices and by DendrisCHIP**^®^** technology and NGS at the Dendris’s laboratory. The method to assess the limit of detection (LOD) is reported in the Supplementary File.

#### 2.1.2. Microbiological Cultures for Bacteria Identification

Samples were transported in sterile containers to the diagnostic laboratory of Centre de Biologie Médicale (CBM), where they were immediately processed. Samples were ground and homogenized (IKA Ultra-Turrax) and 30 µL were inoculated by WASPLab Copan (bioMérieux, Marcy-L’Etoile, France) on appropriate aerobic medium (Columbia + 5%blood agar, Choco-late + PolyViteX agar) and anaerobic medium (Schaedler agar, Thioglycollate Broth) for bacteria identification and Sabouraud Glucose Gelose + Chloramphenicol for yeast and molds identification (media were from Marcy-l’Etoile, bioMérieux, France). Gram staining was performed. Additional details about microbiological cultures are reported in the supplementary file.

### 2.2. Bacterial, Probes Design, and Manufacture of DendrisCHIP^®^OA

A total of 68 probes were designed on the hypervariable region of the 16S rRNA, *IS6110*, and *tuf* genes from the bacteria listed in Table 1. A list of the PCR probes with their target genes and amplicon size is reported in Table S1 in the supplementary file. Multiple alignment analysis using ClustalW (http://www.clustal.org/clustal2/, accessed on 30 November 2021) was applied on the *16S rRNA* gene, which was retrieved from the NCBI database or sequenced prior to creating the probes’ design (see below). The exclusivity of the probes’ sequence was queried against sequences in Genbank database with a BLAST search. Probe quality criteria, namely, length of the oligonucleotide between 20 and 25 nucleotides long, equal melting temperature, lack of hairpin and dimer formation were assessed with Primer 3plus [32,33]. Synthetic oligoprobes were further designed for quality control of the process. The probes were purchased from Eurofins (France) with their 5′ end NH_2_-modified. The DendrisCHIP**^®^**OA manufacturing is described in the Supplementary File.

### 2.3. Process Flow for DendrisCHIP^®^OA Validation

#### 2.3.1. DNA Extraction and PCR

DNA was extracted using DNeasy Blood & Tissue Kit from Qiagen according to the manufacturer’s instructions. The DNA from reference strains was isolated from one colony taken from Petri dish agar plates containing LB medium mixed with 200 µL of DNase-free water. DNA extraction from isolates was carried out according to the type of samples. When they originated from swabs, they were soaked in 200 µL of PBS 1X following a strong shaking on vortex to obtain a bacterial suspension. When they originated from liquid samples, 500 µL was used to extract DNA. The following steps common to all samples were as follows. After centrifugation of samples for 10 min at 3000× *g*, the pellet was resuspended in 180 µL of an enzymatic lysis buffer (lysozyme to 40 mg/mL in 20 mM Tris-Cl, pH 8.0, 2 mM Sodium EDTA, 1.2% Triton^®^X-100) and incubated at 37 °C for 30 min.

Multiplex PCR was performed with a mixture of the 5 primer pairs listed in Table 2 with the reverse primer labeled at its 5′ end with biotin (bio-teg). The PCR reaction was carried out in a total volume of 50 µL in 1X PCR buffer containing 3.75 U of Hot Diamond Taq DNA Polymerase (Eurogentec, Selland, Belgium), 3 mM MgCl2, 0.2 mM deoxynucleotides (dNTPs) (Sigma-Aldrich, St. Louis, MO, USA), and between 0.08 and 0.2 µM of each primer (Integrated DNA Technologies, Clareville, IA, USA) and extracted DNA (between 5 and 100 pg). The amplification was achieved with a PCR in a GTQ-Cycler 96 (Hain Life Sciences, Nehren, Germany) using the following program: 30 s at 94 °C, 30 s at 58 °C, and 40 s at 72 °C for 35 cycles.

#### 2.3.2. Semiautomated Hybridization Process and Reading of the DendrisCHIP^®^OA

PCR purification and hybridization of the purified amplicons on the DendrisCHIP**^®^**OA were performed in a semiautomatic manner using our DendriSTATION. A Microlab starlet (Hamilton, Bonaduz, Switzerland) controlled by VENUS 4.0 equipped with a Hamilton Heater Shaker (HHS, Hamilton, ON, Canada), a Hamilton Heater cooler (HHC, Hamilton, ON, Canada), a HeatPAC ambient 135 °C (Inheco, Planegg, Germany), a NucleoMag SEP (Macherey-Nagel, Düren, Germany), and an HEPA hood (Noroit, Bouaye, France).

The practical condition for hybridization was as follows: heating PCR amplicons (50 µL) for 2 min at 95 °C, addition of hybridization buffer (Denhardt’s solution with 1% Ficoll (type 400), 1% polyvinylpyrrolidone and 1% Bovine Serum Albumin), 2.5× SSC, 100 µg/mL salmon sperm DNA and the 5′bioteg-labelled oligonucleotide complementary of CIH_ol at a final concentration of 1 nM; 125 µL of this mixture was automatically pipetted and loaded in each well of DendrisCHIP^®^OA. The chips were then incubated at 60 °C and 250 rpm for 30 min. The mix was aspirated, and wells were washed once with 200 µL of washing buffer 1 (1X PBS). Then, 100 µL of diluted HRP-streptavidin was added and incubated in the dark for 20 min. The solution was aspirated, and wells were washed three times with 200 µL of washing buffer 2 (1× PBS, 0.05% Tween-20). In the last step, 100 µL of sciCOLOR T3 substrate (Scienion, Berlin, Germany) was added automatically into the wells and incubated again for 20 min in the dark. The substrate was removed, and the chips were dried at 50 °C for 5 min and then left at room temperature for another 5 min. The DendrisCHIP^®^OA were read using the sciREADER CL2 (Scienion Inc., Berlin, Germany) reader. Results were compiled in xlsx spreadsheets. For statistical analysis, the raw data for each spot was subtracted from its surrounding background and the median of the triplicate spots, which corresponded to one probe, was used.

#### 2.3.3. Data Treatment Using Machine-Learning Methods and Statistical Analysis

The first step in order to apply a learning method is to build a training set. Here, it consists of hybridization signals obtained from 619 samples from pure bacterial strains, mixed strains, isolates with known bacteria, as well as spiked with pure bacterial strains. In this training set, the targets to be predicted are those presented in Table 1 (n = 20) and the variables are the designed probes’ hybridization signals. It is noteworthy that there are relationships between the different targets: species belonging to a family/genus were labeled for both the family/genus and the species (e.g., *Staphylococcus aureus* must to be detected as *Staphylococcus aureus* and *Staphylococcus* spp. simultaneously). Moreover, samples may contain more than one pathogen. Therefore, we wanted to solve a multilabel detection problem since it is necessary to be able to predict several labels at the same time. A naive strategy to solve this multilabel problem is to transform it on binary classification problems. Usually, the random forest (RF) method [34] is used to predict the presence of each bacteria and an aggregation of the single predictions is applied. Random forest is a supervised learning method based on multiple decision trees. It aggregates the predictions of each independent tree and creates a probability of the presence of bacteria in the sample. This method has the advantage of providing an indication of the importance of the probes in the model so it allows to determine the probes useful for the discrimination of each pathogen. In our context, this method does not take into account the multilabel and hierarchical aspect of targets. After a comparative study of the learning algorithms that can deal with this problem, the hierarchy of multilabel classifier (HOMER) [35] combined with the multilabel version the random forest proposed by [36] turned out to be the most performant. This retained strategy is implemented in the R package *utiml* [37].

The main idea of the HOMER algorithm is to transform the multilabel classification task into a tree-shaped hierarchy of simpler multilabel classification tasks. For this purpose, it alternates the two following steps to construct a hierarchy of multilabel classifiers: (i) creates K metalabels using the balanced K-means algorithm (clustering of the targets at each node of the hierarchy) and (ii) constructs multilabel classifiers (obtained with random forest in our work) to predict metalabels. This procedure is implemented using the function *homer* from *utiml* with default parameters for clustering (clusters with K = 3, method = balanced) and with parameter “base.algorithm” RF for the learning. The prediction was computed with the function *predict*. Then, to determine algorithm performance, cross validation was performed while using the leaves one data point out (LOOCV) method on the database. The function multilabel_evaluate with parameter measure = ”all” was used to obtain the accuracy, F1 score, and hamming loss.

The confusion matrix was computed to obtain true positives (TP), true negatives (TN), false positives (FP), and false negatives (FN) with the function multilabel_confusion_matrix (from utiml see [37]). Sensitivity is defined as the probability of correctly detecting the presence of a bacterium calculated as TP/TP + FN. Specificity is the probability of correctly rejecting an absent bacterium calculated as FN/FP + TN.

(1) $$\mathrm{Sensitivity}\;(\%)\;\pm \;1.96\times \sqrt{\left(1-\mathrm{sensitivity}\right)*\left(\mathrm{sensitivity}\right)/n1\;};\;\mathrm{with}\;n1=\mathrm{TP}+\mathrm{FN}$$

(2) $$\mathrm{Specificity}\;(\%)\;\pm \;1.96\times \sqrt{\left(1-\mathrm{sensitivity}\right)*\left(\mathrm{sensitivity}\right)/n0};\;\mathrm{with}\;n0=\mathrm{FP}+\mathrm{TN}$$

as n1/n0 were for some bacteria lower than 30, the 95% confidence interval for sensitivity and specificity was calculated using proportion_confint function with parameter method = “beta” from Python library statsmodel according to [38].

### 2.4. Process Flow with Next-Generation Sequencing (NGS) Targeted on 16S rRNA Gene

Samples were sequenced using an Illumina iSeq 100 instrument (Illumina Inc., San Diego, USA) with paired-end reads at 150 cycles. Bacteria V3 and V4 regions in the *16S rDNA* gene were amplified with primer Fwd (TCGTCGGCAGCGTCAGATGTGTATAAGAGACAGCCTACGGG-NGGCWGCAG) and Rvd (GTCTCGTGGGCTCGGAGATGTGTATAAGAGACAGGAC-TACHVGGGTATCTAATCC). Libraries were prepared using Nextera XT Index Kit v2 (Illumina, Évry-Courcouronnes, France) and quantified with KAPA SYBR FAST qPCR (Roche, Boulogne-Billancourt, France). Metagenomic analyses were performed using Illumina pipeline to remove adapter sequences and filter out residual human and PhiX sequences. The remaining paired-ends reads were assembled into tags in FlAsH v.1.2.7, clustered into operational taxonomic units (OTUS) at 97% similarity using sparse v7.0.1001 (http://drive5.com/uparse/, accessed on 10 January 2022), and identified with respect to genus and species using Ribosomal Database Project (RDP) classifier v2.2 against sIlvA (v128), green genes (13_8), and NCBI databases.

## 3. Results

### 3.1. Construction and Validation of the DendrisCHIP^®^OA

The pathogens potentially present in osteoarticular infections are quite numerous, including commensal organisms living on the skin that can accidentally infect implantable biomedical devices. In order to adapt our DendrisCHIP^®^ technology for diagnosing this type of infection, we defined a panel of bacteria that have already been shown to be implicated in PJI. A consensus of 20 bacteria was therefore proposed from a literature survey and with the approval of the INOVIE group (https://inovie.fr/), which is a major player in France for medical diagnosis. As indicated in Table 1, the selected bacteria were designated at the family (e.g., *Enterobacteriaceae*), genus (e.g., *Neisseria*), and species (e.g., *Staphylococcus aureus*) level. This stratification should allow a large degree of completeness in identifying pathogenic bacteria. To be able to detect these bacteria, we firstly designed a large set of oligoprobes in the hypervariable region of the 16S rRNA gene. In addition, for the identification of *Enterobacteriaceae* and *Staphylococcus* at the genus and species level, additional probes were designed on the *tuf* gene encoding the elongation factor Tu as this gene was reported to be more discriminatory than the *16S rRNA* gene to identify these bacteria in clinical isolates [38]. Oligoprobes in the *IS6110* gene was also designed for the identification of *M. tuberculosis* [39,40]. Finally, we considered oligoprobes on *mecA* to identify potential β-lactam resistant bacteria from the *Staphylococcus* genus [41]. It is worth noting that all PCR amplicons in the target genes had similar sizes and hybridization temperature, enabling us to standardize the conditions for multiplex PCR and hybridization on the chips. A total of 68 probes were deposited in triplicates on DendriSLIDE^®^ by the Scienion SCIFLAXARRAYER SX to generate the corresponding DendrisCHIP^®^OA. The whole configuration of the DendrisCHIP^®^OA can be found in Figure S1 in the supplementary file.

As extensively described in a previous report [25], the initial step in the validation of the DendrisCHIP^®^OA was to ensure that the oligoprobes exhibited a positive hybridization signal with their corresponding targets. It is also worth noticing that the colorimetric detection of hybridization signals turned out to be as sensitive as the fluorescence method used in our previous report, but more importantly, the hybridization signals with this new labeling and reading procedure were extremely stable over time, as same intensity values could be recorded even one month after the first reading. Next, we had to assign a specific signature for each bacteria, enabling us to construct a database, which was built from a total of 619 samples. These samples were obtained from pure bacterial cultures, mixed cultures, spiked bacteria in microbial negative isolates, as well as isolates that contained at least one well-certified bacteria by microbial cultures. The distribution between these different origins can found in Figure S2 in the supplementary file. Accordingly, 45% of the hybridization signals were obtained from bacteria in isolates, which integrates the matrix effects notably due to the presence of possible PCR inhibitors as well as the large amount of nonbacterial DNA. In addition, this wide variety of sampling may explain the variability in absolute intensity values for each bacterium (see Figure S3 in the supplementary file), which reflects the difference in abundance of bacteria in the samples. We also took into account that a sample could contain several bacteria and that a bacterium can be assigned as a family, a genus, or a species, which is a typical problem of hierarchical multilabel classification that can be addressed by the hierarchy of multilabel classifier (HOMER) [35] combined with the multilabel version of the random forest proposed by [36]. Application of this algorithm enables us to assign to each bacterium a specific signature with a probability value of sensitivity and specificity reported in Table 2.

While the specificity, which is the probability to correctly reject the absence of bacteria in a sample, was in the range of 95–98%, the sensitivity, which is the probability to correctly detect a positive bacterium in a sample, was more dispersed among the bacteria. The dispersion value in sensitivity accompanied by a high width of 95% of CI can be explained by several reasons, including insufficient sample size, lack of probe specificity, technical limitations such as the quality and quantity of the extracted DNA and the efficiency of the multiplex PCR. As a final step in the validation of our DendrisCHIP^®^OA, we estimated the limit of detection (LOD) by hybridizing the amplicons obtained by PCR on serial dilutions of pure DNA of some of the bacteria listed in Table 1. A LOD in the range of 10^3^ CFU/mL was estimated for *Mtu, Ngo*, *Ssp*, *Sau,* and *Sag*, whereas it was one log higher for *Ent*, *Msp*, *Nsp,* and *Spn*. Overall, the LOD was in the same range as previously reported for bacteria implicated in respiratory disease [25].

### 3.2. Isolates Analyzed by DendrisCHIP^®^OA and Comparison with Microbial Cultures

**Figure 1:**
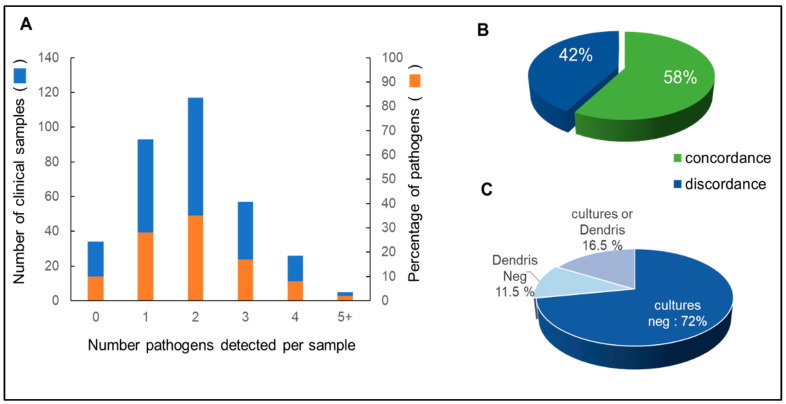
Detection of pathogen bacteria in isolates by DendrisCHIP^®^OA and microbial cultures. In (**A**) is represented the number of pathogens per sample as detected by DendrisCHIP^®^OA. In (**B**) is shown the concordance and discordance in the detection of bacteria by DendrisCHIP^®^OA and by the microbial cultures. In (**C**) is reported the distribution of the discordant results with respect to the detection by microbial culture and by DendrisCHIP^®^OA. Culture Neg = negative by microbial culture/positive by DendrisCHIP^®^OA; Dendris Neg = negative by DendrisCHIP^®^OA/positive by microbial cultures; culture or Dendris = either one or the other as negative or positive.

A total of 462 analyses were performed using DendrisCHIP^®^OA from 333 isolates, as approximately 25% were repeated at least twice. The results obtained with our technology showed that in only 10% of the samples (34 out of 333) no bacteria were detected, and in the remaining 90%, 1 to 5 bacteria could be detected per sample, with a peak of 2 bacteria per sample in 40% of the total samples (Figure 1A). This result is in agreement with other molecular biology techniques that often detect more than one bacterium in bone and joint infections [13,42,43].

These 333 samples were also analyzed by microbial cultures carried out independently by clinical laboratories according to their internal protocol described in Materials and Methods. The microbial data were compared with our results in terms of concordance and discordance, assuming that concordance between the two methods was assigned when at least one bacterium was detected in both methods, regardless of genus or species, whereas discordance occurred in all other situations. As indicated in Figure 1B, only 58% of samples were concordant between the two methods. The 42% discordance could be explained by the fact that 72% of the tests were negative for microbial cultures, but only 11.5% were negative with DendrisCHIP^®^OA. These data support the general trend that microbial cultures result in a higher frequency of false negatives than molecular methods [4,10,12].

### 3.3. Reliability of DendrisCHIP^®^OA Technology in Diagnosing Osteoarticular Infection by Comparison with NGS

**Figure 2:**
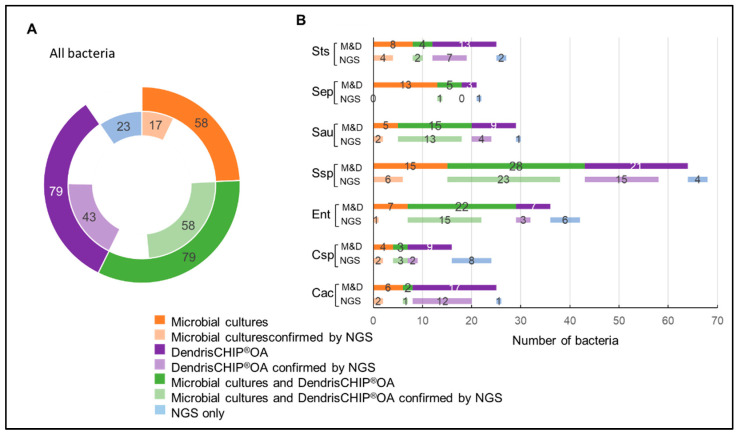
Comparison between molecular methods and microbial cultures for identification of bacteria in isolates. A total of 101 samples were sequenced, giving rise to a total of 141 identified bacteria by NGS according to criteria defined in Materials and Methods. The identified bacteria were compared with those identified by DendrisCHIP^®^OA and microbiological methods in (**panel A**). Comparison at the level of single bacteria species between the three methods is reported in (**panel B**). The abbreviation for bacteria can be found in Table 1.

In order to better evaluate the reliability of our DendrisCHIP^®^ technology, not only in terms of capacity to accurately detect bacteria but also in terms of correct identification of bacteria in isolates, we decided to sequence 101 isolates taken at random from our collection. The NGS data were obtained by sequencing *16S rRNA* gene amplicons according to the Illumina iSeq protocol. As this method is expected to be extremely comprehensive, we selected the bacteria that fell within the 75% of total reads corresponding to identified bacteria and considered them as the reference for comparison with the data obtained by microbiological culture and by DendrisCHIP^®^OA. In addition, only bacteria of the panel in Table 1 were taking into consideration for the comparison. From the sequencing of 101 isolates, a total of 141 bacteria were identified by NGS (Figure 2A). As indicated in this figure, 32% (17/58) of the bacteria identified by microbial cultures were confirmed by sequencing, whereas 55% (43/79) identified by DendrisCHIP^®^OA matched those of NGS.

The concordance between NGS and DendrisCHIP^®^OA was also indicated by performing a comparative analysis at the level of each identified bacterial species (Figure 2B). For example, no *S. epidermidis* identified by microbiology was confirmed by sequencing. On the contrary, 12 of the 17 *Cutibacterium acnes* (Cac) species identified by DendrisCHIP^®^OA were found by NGS. It is also shown in Figure 2B that the most represented bacteria in isolates was *Staphylococcus* spp (56% of the samples), followed by *Enterobacteriaceae* spp. (Ent), *Streptococcus* (Sts), and *Cutibacterium acnes* (Cac). This data was not unexpected since these are the bacteria the most often identified in PJI [19]. Overall, this comparative analysis clearly indicated that the identification of pathogenic bacteria by microbial method did not correspond well to that obtained by sequencing, unlike the DendrisCHIP^®^ technology. This better concordance between NGS and DendrisCHIP^®^OA can be further illustrated by linear regression taking the data from NGS as the model, allowing to estimate a R^2^ of 0.93 with DendrisCHIP^®^OA that slightly decreased to 0.89 when bacteria were identified only by NGS whereas the R^2^ was in the range of 0.6 with microbial cultures (see Figure S4 in supplementary file).

The reliability of the DendrisCHIP^®^OA to identify bacteria in isolates was further consolidated by the fact that among the 19 samples that were negative with this method, 65% of them were confirmed by NGS, whereas 15 of these “negatives” turned out to be positive with microbial cultures (Figure S5 in supplementary file). It should be noted that the bacteria identified in these positive microbial cultures were *Streptococcus* spp. that primarily colonize skin, mucous membranes, and throat. In contrast, from the 18 negative samples assessed by microbial culture, 14 turned to be positive with DendrisCHIP^®^OA and 16 with NGS. The bacteria identified in these samples were mostly *Cutibacterium acnes* and *Staphylococcus* spp. (Figure S5 in supplementary data).

**Figure 3:**
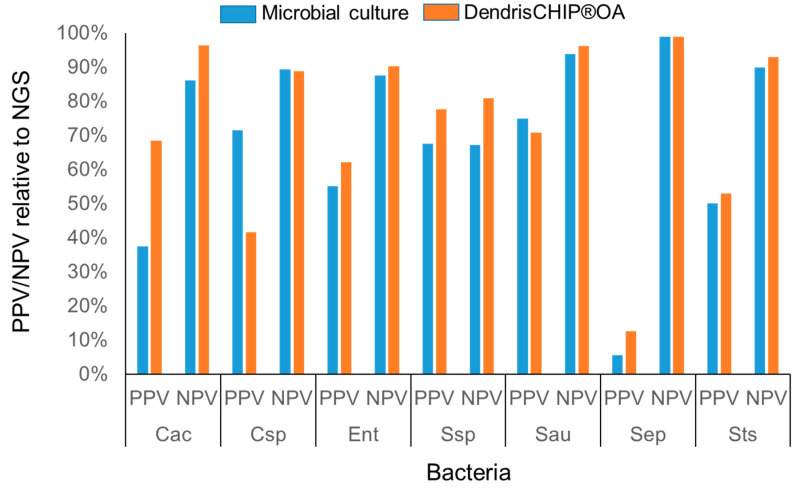
Evaluation of the predictive positive and negative values (PPV; NPV) of diagnostic tests by DendrisCHIP^®^ technology and microbial cultures relative to NGS data obtained from 101 isolates.

Finally, taking NGS data as the reference for bacteria identification in samples, we estimated the performance characteristic of the two diagnostic methods, namely, microbial culture and DendrisCHIP^®^OA technology, by calculating the positive predictive value (PPV) which indicates the probability that the bacteria are actually present when the test result is positive, and the negative predictive value (NPV) which is the probability that the bacteria are absent when the test result is negative [44]. As shown in Figure 3, the PPV for most of the bacteria was consistently better with DendrisCHIP^®^OA than with microbial cultures, while the NPV was roughly similar for both methods. These data indicate that the DendrisCHIP^®^ technology is more able to detect and identify bacteria in samples than the microbiology.

## 4. Discussion

A revolution in the diagnosis of bacterial and viral infections is taking place, triggered in particular by the COVID-19 pandemic, with the penetration of molecular methods even in simple medical office, allowing a genotype-based diagnosis, which should be intrinsically more accurate and rapid than phenotypic diagnosis based on conventional microbial cultures. However, the transition to molecular syndromic-based diagnosis, i.e., allowing the detection and identification of all possible pathogen(s) responsible for an infection [45,46,47] without any preconceived ideas and in a single patient sample, is still paved with numerous technical and economic difficulties. This work was dedicated to evaluating the potential of our innovative DendrisCHIP^®^ technology to detect and identify bacteria commonly implicated in osteoarticular infections, which are painful and disabling diseases that are particularly difficult to diagnose [10,12]. We already described in a previous report that the novelty of our technology was to unlock two major limitations of the classical DNA microarrays technique, namely, to provide a higher signal to noise ratio and hence higher accessibility of nucleic acid targets to the probes by functionalization of the glass slides with dendrimers. The second and critical advancement of our technology was to develop a specific decision algorithm based on machine-learning methods. In this present work, we have upgraded our initial algorithm by implementing the hierarchy of multi-label classifier (HOMER) [35], taking into account that more than one bacterium (label) can be identified in a sample and that there is a hierarchical structure in the label (e.g., family, genus, species). However, the power of our decision algorithm and, henceforth, the accuracy of the diagnostic in terms of sensitivity and specificity strongly rely on the size of the training database, and on the origin of samples to build this database, meaning, here, that it is critically important to have an equilibrated representation of all potential pathogens in the database. The specificity is mainly affected by the high representativeness of the different bacteria in the database while the sensitivity will be mainly dependent on the types of samples, especially the signal on the chip depends on the quality and quantity of the DNA extracted from the biological samples and the efficiency of the multiplex PCR performed on these samples before hybridization. In this present work, we have therefore taken care of these problems by building a training database in which 50% of the data (as hybridization signals) arose from isolates or pure bacteria spiked in isolates.

The central part of this study was to evaluate the reliability of our technology, firstly, by comparing the results of bacteria detection with routine microbial cultures, which were carried out independently by pathologists. This comparative analysis revealed a concordance of 58% between the two methods. More importantly, a great part of the 42% of discordance between the two methods was due to a very high proportion of culture-negative tests (72%) that turned out to be positive with the DendrisCHIP^®^OA (58% of the 72%). We therefore compared these data obtained with our technology with the NGS targeted on 16S rRNA gene amplicons and also used NGS as a reference method to validate the reliability of our technology in terms of identification of bacteria. Our results showed a very good concordance between the two molecular methods, which was in the range of 90%, whereas it was only 60% between NGS and the microbial cultures. One should note that NGS revealed more microbial species than DendrisCHIP^®^OA, which is limited to those in Table 1. On the other hand, the fact that microbiology was less concordant than molecular technologies can be attributed to several causes, including culture time, biases related to the collection of a few colonies on a Petri dish for phenotypic identification that is dependent on the technical operator microbes that cannot grow under these culture conditions or that are missing from the mass spectrometer database, as well as that some samples arose from patients treated with antibiotics. An additional finding using DendrisCHIP^®^ technology was to identify more than one bacterium per sample in more than 40% of the isolates. This result is also consistent with other molecular technologies [19,20,21], assuming that the infection may be more often polymicrobial in nature than anticipated [11]. However, these results must be taken with caution because polymicrobial infections can be due to contaminations during the sampling procedures, which explain the many precautions that must be taken to avoid misconceptions and errors in PJI diagnostics [8].

**Figure 4:**
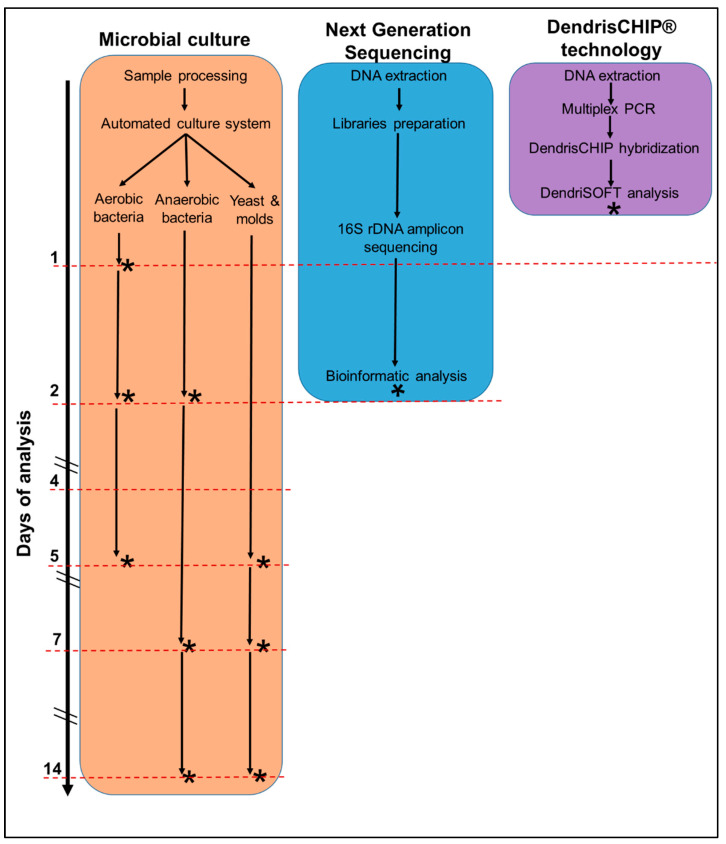
Time scale for the workflow of the microbial culture, NGS, and DendrisCHIP^®^ technology in PJI diagnostics. The asterisk indicate the time at which a diagnostic result can be provided.

The microbial culture is, to date, the conventional standard reference in many practices and in particular in bone and joint infections diagnostic. However, as schematically depicted in Figure 4, and regardless of the inadequacy of this method especially with biofilms or slow-growing, antibiotic-tolerant subpopulations [2,48], microbial culture is extremely complex, time-consuming, and very labor-intensive.

Comparative to that, the NGS technology is much faster as, overall, it will take 2 days to deliver the results, but the power of this method is also its Achilles heel as all the bacteria that can be identified by this power method might not be relevant to the disease but endogenously present or coming from contaminating tools used during surgical intervention. On the other hand, the DendrisCHIP^®^ technology fulfills several of the clinical microbiology criteria as it is an easy-to-use method (a laboratory technician can handle the technology in half a day), it addresses the syndromic approach of diagnostics, and delivers results in less than one day. In addition, this technology will avoid the NGS trap since our DendrisCHIP^®^OA will carry only probes that will identify solely bacteria that have been acknowledged by the clinical/medical community as truly involved in a given infection disease, such as PJI.

In conclusion, the DendrisCHIP^®^ technology is an innovative molecular technique for syndromic diagnostic that presents several technical features matching the clinical microbiology expectation. Although we have presented, in a previous report [25], some limitations of this technology (i.e., semiquantitative method; detection of bacteria while dead, which is the major problem of any molecular tool using DNA) and solved some of them (i.e., semiautomation of the workflow to limit external contamination), a decisive step for its approval in clinical laboratories will be to carry out a multicenter study involving a large collection of isolates with well-classified patients who have undergone primary and revision joint replacement surgery. In this study, the results will have to be analyzed against the clinical diagnostic criteria established by the MSIS and EBJIS consortia methods [7,49] in order to conclude on the reliability and robustness, combined with the ease of use of the DendrisCHIP^®^ technology. In addition, this study could serve to define the cut-off between clear infection, the “grey zone”, and clear negative results in this very complex infection disease.

## Figures and Tables

**Table 1:** List of bacteria usually implicated in PJI and targeted by the DendrisCHIP^®^OA.

Bacteria	Abbreviation Used in This Work	Taxonomy	Gene	Accession Number
*Enterobacteriaceae*	Ent	family	16S rRNA	/
*Enterobacter cloacae*	Ecl	species	16S rRNA	KC990822.1
*Escherichia coli*	Eco	species	16S rRNA	NR024570.1
*Klebsiella pneumoniae*	Kpn	species	16S rRNA	KC99081717.1
*Proteus mirabilis*	Pmi	species	16S rRNA	MN689880.1
*Corynebacterium* spp.	Csp	genus	16S rRNA	LT960557.1; NR119182.1; KF564647.1
*Cutibacterium acnes*	Cac	species	16S rRNA	DQ672261.1
*Enterococcus faecalis*	Efa	species	16S rRNA	AB362602.1
*Mycoplasma* spp.	Msp	genus	16S rRNA	/
*Mycoplasma pneumoniae*	Mpn	species	16S rRNA	AF132741.1
*Mycoplasma genitalium*	Mge	species	16S rRNA	NR026155.1
*Neisseria* spp.	Nsp	genus	16S rRNA	/
*Neisseria gonorrhoeae*	Ngo	species	16S rRNA	AM921674.1
*Neisseria meningitidis*	Nme	species	16S rRNA	NR104946.1
*Mycobacterium tuberculosis*	Mtu	species	IS6110	Y14045.1
*Kingella kingae*	Kki	species	16S rRNA	AY628416.1
*Staphylococcus* spp.	Ssp	genus	16S rRNA *tuf*	/
*Staphylococcus aureus*	Sau	species	16S rRNA *tuf*	DQ630753.1 AF298796.1
*Staphylococcus epidermidis*	Sep	species	16S rRNA *tuf*	NR036904.1 AF298800.1
*Staphylococcus warneri*	Swa	species	16S rRNA *tuf*	LN998066.1 AF298806.1
*Staphylococcus haemolyticus*	Sha	species	16S rRNA *tuf*	LN998078.1 AF298801.1
*Staphylococcus hominis*	Sho	species	16S rRNA *tuf*	HG941670.1 AF298802.1
*Staphylococcus lugdunensis*	Slu	species	16S rRNA *tuf*	NR024668.1 AF298803.1
*Streptococcus* spp.	Sts	genus	16S rRNA	/
*Streptococcus agalactiae*	Sag	species	16S rRNA	LC545464.1
*Streptococcus pyogenes*	Spy	species	16S rRNA	NR028598.1
*Streptococcus pneumoniae*	Spn	species	16S rRNA	NR028665.1

**Table 2:** Sensitivity and specificity for each bacterium detected on the DendrisCHIP^®^OA determined as described in Materials and Methods.

Bacteria	Sensitivity (%)	Specificity (%)	CI95 Sensitivity (%)	CI95 Specificity (%)
*Cutibacterium acnes*	95.8	98.4	86–99	97–99
*Corynebacterium* spp.	82.6	98.1	69–92	97–99
*Enterobacteriacea*	96.9	98.4	92–99	97–99
*Enterobacter cloace*	60.0	98.8	36–81	98–100
*Escherichia coli*	85.2	99.2	66–96	98–100
*Klebsiella pneumoniae*	92.9	99.2	76–99	98–100
*Proteus mirabilis*	93.8	99.7	70–100	99–100
*Enterococcus faecalis*	92.1	99.8	79–98	99–100
*kingella kingae*	78.9	99.5	54–94	99–100
*Mycobacterium tuberculosis*	97.0	100.0	84–100	99–100
*Mycoplasma* spp.	75.0	100.0	59–87	99–100
*Mycoplasma genitalium*	90.0	99.7	73–98	99–100
*Mycoplasma pneumoniae*	72.7	100.0	39–94	99–100
*Neisseria* spp.	86.8	99.5	72–96	98–100
*Neisseria gonorrhoeae*	76.2	98.8	53–92	98–100
*Neisseria meningitidis*	44.4	99.5	22–69	99–100
*Pseudomonas aeruginosa*	83.3	99.5	59–96	99–100
*Staphylococcus* spp.	94.1	96.4	90–97	94–98
*Staphylococcus aureus*	93.1	98.0	87–97	96–99
*Staphylococcus epidermidis*	74.3	99.8	57–88	99–100
*Staphylococcus haemolyticus*	69.2	100.0	39–91	99–100
*Staphylococcus hominis*	91.7	100.0	62–100	99–100
*Staphylococcus lugdunensis*	81.3	99.8	54–96	99–100
*Staphylococcus warneri*	92.3	99.8	64–100	99–100
*Streptococcus* spp.	94.0	97.6	87–98	96–99
*Streptococcus agalactiae*	93.3	99.8	78–99	99–100
*Streptococcus pneumoniae*	96.0	99.3	80–100	98–100
*Streptococcus pyogenes*	76.9	100.0	46–95	99–100

## Supplementary Materials

The following supporting information can be downloaded at: https://www.mdpi.com/article/10.3390/diagnostics12061353/s1, supplementary file which contains additional Materials and Methods; Table S1: List of PCR primers for amplification of target genes; Figure S1: Scheme of the DendrisCHIP^®^OA manufacturing; Figure S2: Composition of the training database; Figure S3: Intensity of the oligoprobes designed for each bacterium in the panel in Table 1 on the DendrisCHIP^®^OA expressed as boxplots; Figure S4: Linear regression analysis of identified bacteria in isolates by DendrisCHIP^®^OA and microbial cultures against bacteria identified by NGS as the model; Figure S5: Identification of bacteria in isolates by NGS which are negative for microbial culture (A) or negative with the DendrisCHIP^®^OA (B). Supplementary File also contained Ref [49,50,51] given in References List in the main text.
