# Supplementary material for: The DendrisCHIP® Technology as a New, Rapid and Reliable Molecular Method for the Diagnosis of Osteoarticular Infections
Source: Diagnostics (Basel). 2022 May 30;12(6):1353. doi: 10.3390/diagnostics12061353 (PMC9222036; doi:10.3390/diagnostics12061353)
Supplement: Supplementary file 1 [file diagnostics-12-01353-s001.zip › diagnostics-1715794-supplementary.pdf]

Supplementary Materials: The DendrisCHIP® technology as a new, rapid and reliable molecular method for the diagnosis of osteoarticular infections

Elodie Bernard [1], Thomas Peyret [1], Mathilde Plinet [1], Yohan Contie [1], Thomas Cazaudarré [1], Yannick Rouquet [2], Matthieu Bernier [2], Stéphanie Pesant [1], Richard Fabre [2], Aurore Anton [1], Cathy Maugis-Rabusseau [3] and Jean Marie François [1,4,*]

[1] Dendris SAS, 335 rue du Chêne Vert, F-31670 Labège, France; ebernard@dendris.fr (E.B.); tpeyret@dendris.fr (T.P.); mplinet@dendris.fr (M.P.); ycontie@dendris.fr (Y.C.); tcazaudarre@dendris.fr (T.C.), spesant@dendris.fr (S.P.), aanton@dendris.fr (A.A.)
[2] Laboratoire CBM-Inovie, F-31000 Toulouse, France; yannick.rouquet@inovie.fr (Y.R.); matthieu.bernier@inovie.fr (M.B.); richard.fabre@inovie.fr (R.F.)
[3] Institut de Mathématiques de Toulouse, UMR5219, Université de Toulouse, CNRS, INSA, F-31077 Toulouse, France; cathy.maugis@insa-toulouse.fr (C.M.R.)
[4] TBI, Université de Toulouse, CNRS, INRAE, INSA, 135 Avenue de Rangueil, F-31077 Toulouse, France
* Correspondence: fran_jm@insa-toulouse.fr

Additional Materials and Methods

Assessment of detection limit

To assess the limit of detection (LOD) of our technology, pure bacterial DNA from *Escherichia coli* used as *Enterobacteriaceae* family, *Mycobacterium tuberculosis*, *Mycoplasma genitalium*, *Neisseria meningitidis* and *gonorrhoeae* (used as *Neisseria spp*), *Staphylococcus aureus* and *Streptococcus agalactiae* was purchased from Orgentec SASU (Trappes, France). The quantified DNAs had a concentration between 1.3 to 2 x 108 copies/µL PCR was performed on the appropriate DNA target gene using the primers pair (Table 2) on serial dilution of pure DNA and the labelled amplified targets were hybridized on DendrisCHIP®. To obtain statistical data, this experiment was repeated 10 times with the same serial dilution.

Microbiological cultures for bacteria identification

THIO liquid media were incubated at 35 °C and inoculated on COS and SCS medium after 2 and 7 days of incubation and then inspected for growth 5 days later. Solid media were incubated at 35 °C +5% CO2 or in jar or bag for anaerobic media. Cultures on SGC media were incubated first at 35 °C +5% CO2 during 48 h and then 72 h at 30 °C in standard atmosphere. Aerobic media were inspected for growth on the first and the 2nd day of incubation; anaerobic media were inspected on the 2nd and 14th day of incubation. Cultures on SGC media were inspected on the 5th day of incubation. When growth was observed on plates, identification was further performed on a Biotype MALDI-TOF mass spectrometry instrument (Microflex, Bruker, Bremen, Germany). Antibiogram in liquid medium were performed on VITEK 2 and systematic complement for daptomycine and quinupristine were done for *Staphylococcus spp* on standard MHE gelose ATB (Mueller Hinton BioMérieux, Marcy L'Etoiled, France). Pathogenic microbial strains were cryopreserved according to the manufacturer's instructions (AEB 400100 cryobeads, BioMérieux, marci l'Etoile, France) and stored at -80 °C.

Probes design to make the DendrisCHIP®OA

List of the PCR probes with their target genes and amplicon size is given in Table S1. Multiple alignment analysis using ClustalW (http://www.clustal.org/clustal2/) was ap-

plied on the *16S rRNA* gene, which has been retrieved from NCBI database or were sequenced prior to make probes design (see below). The exclusivity of the probes sequence was queried against sequences in Genbank database with a BLAST search. Probe quality criteria, namely length of the oligonucleotide between 20 and 25 nucleotides long, equal melting temperature, lack of hairpin and dimer formation were assessed with Primer 3plus [32,33]. Synthetic oligoprobes were further designed for quality control of the process. The probes were purchased from Eurofins (France) with their 5'end NH_2-modified.

Table S1. List of PCR primers for amplification of target genes.

Name	Sequence 5' - 3'	Tm °C	%GC	Target Gene [Accession Number]	Amplicon Size (bp)	Ref.
16S-F osteo	GCAGCCGCGGTAATAC	61	62.5	*16S rRNA*	510	[13]
16S-R osteo	CACGAGCTGACGACA	56.5	60			
MT2-F	TCCAGCGCCGCTT	62.2	69.2	*IS6110*	204	[49]
MT1-R	CAGATGGCTTGCTCGAT	60.7	52.9	[Y14045.1]		[40]
staph-F	CCGTGAACGTGGTCAA	60.9	56.3	*tuf*	370	[50]
staph-R	ACCATTTCAGTACCTTCTGG-TAA	60.8	39.1			
β-Globin-F	GAA-GAGCCAAGGACAGGTAC	60.8	55	*β-globin* [UO1317.1]	407	Den-dris
β-Globin-R	GGAAAATAGACCAA-TAGGCAG	59.6	42.9			
MecA-MR3-F	AAATCGATGGTAAAGGTTGG	60.7	40.9	*mecA*	533	[51]
MecA-MR4-R	AGTTCTGCAGTACCGGATTT	60.7	47.8	[NG047936.1]		

DendrisCHIP®OA manufacturing

The DendrisCHIP® bearing the oligoprobes of the targeted bacteria implicated in PJI was termed DendrisCHIP®OA. They were manufactured essentially as described in Trevisiol et al. [30] using oligoprobes at a concentration of 50 µM in 0.2 M phosphate buffer pH 9.0 and printed in triplicate on DendriSLIDE® by the piezo electrical dispensing with the sci-FLEXARRAYER SX robot from Scienion (Berlin, Germany). The average diameter size of the spots was 180 µm ± 10 µm and each spot was spaced by 400 µm. Five additional oligoprobes were included in this pattern for quality control (QC) purpose. A first type was a 25- mer synthetic probe enabling the positioning of the DendrisCHIP®OA during the image processing. A second kind was a 25-mer synthetic probe for validation of the hybridization step. Finally, a 25-mer oligoprobe was designed to hybridize with a 407 bp fragment of human *β-globin*gene, which was used as an internal control of the PCR amplification carried out on the extracted DNA samples. The whole configuration of the DendrisCHIP®OA is shown in Figure S1. Up to 120 DendriSLIDE® can be processed on the sci-ARRAYER platform at once in 35 h, with each slide bearing sixteen DendrisCHIP®OA. These DendrisCHIP®OA were separated each to other by a custom plastic structure (MPM, Muret, France). At the end of the spotting process, the DendriSLIDE® were plunged in a bath containing 1.74 g/L of $NaBH_4$ in milliQ water for 30 minutes followed by three washing in water for 5 minutes each and then dried by centrifugation at 900 rpm for 30 minutes.

REF	Kki	Cac	MecA	Nme	REF	Kki	Cac	MecA	Nme	REF	Kki	Cac	MecA	Nme
2	Mtu	Cac	MecA	Nsp	2	Mtu	Cac	MecA	Nsp	2	Mtu	Cac	MecA	Nsp
Ssp	Mtu	Cac	MecA	Nsp	Ssp	Mtu	Cac	MecA	Nsp	Ssp	Mtu	Cac	MecA	Nsp
Eco	Mtu	Cac	MecA	Nsp	Eco	Mtu	Cac	MecA	Nsp	Eco	Mtu	Cac	MecA	Nsp
Eco	Mtu	Efa	Pae	Sag/Spy	Eco	Mtu	Efa	Pae	Sag/Spy	Eco	Mtu	Efa	Pae	Sag/Spy
Pmi	Sep	Efa	Kpn	Sag	Pmi	Sep	Efa	Kpn	5CIP	Pmi	Sep	Efa	Kpn	Sag
Pmi	Sep	Ssp	Ecl	Mpn	Pmi	Sep	Ssp	Ecl	6CIP	Pmi	Sep	Ssp	Ecl	Mpn
Mpn	Sha/Swa	Efa	Ecl	Mpn	Mpn	Sha/Swa	Efa	Ecl	7CIP	Mpn	Sha/Swa	Efa	Ecl	Mpn
Mpn	Csp	Bce	Kpn	Mpn	Mpn	Csp	Bce	Kpn	1CIH	Mpn	Csp	Bce	Kpn	Mpn
Mge	Sep	Swa	Pae	Mpn	Mge	Sep	Swa	Pae	REF	Mge	Sep	Swa	Pae	Mpn
Ngo	Cac	Sts	Sau	5CIP	Ngo	Cac	Sts	Sau	Sag	Ngo	Cac	Sts	Sau	5CIP
Ngo	Ecl	Sag	Spn/Spy	6CIP	Ngo	Ecl	Sag	Spn/Spy	Mpn	Ngo	Ecl	Sag	Spn/Spy	6CIP
Kpn	Csp	Nsp	Spn/Spy	7CIP	Kpn	Csp	Nsp	Spn/Spy	Mpn	Kpn	Csp	Nsp	Spn/Spy	7CIP
Kki	Sho/Slu	Nsp	Spn	1CIH	Kki	Sho/Slu	Nsp	Spn	Mpn	Kki	Sho/Slu	Nsp	Spn	1CIH
REF	Efa	146	Spn	REF	REF	Efa	146	Spn	Mpn	REF	Efa	146	Spn	REF

Figure S1. Scheme of the DendrisCHIP®OA. The location of oligoprobes for each bacteria as indicated by their abbreviated name (see Table 1 for correspondence to the complete name) on the DendriSLIDES®, together with the probes for quality control refereed as CIP and internal reference oligoprobes that correspond to *β-globin* gene and is refereed as REF.

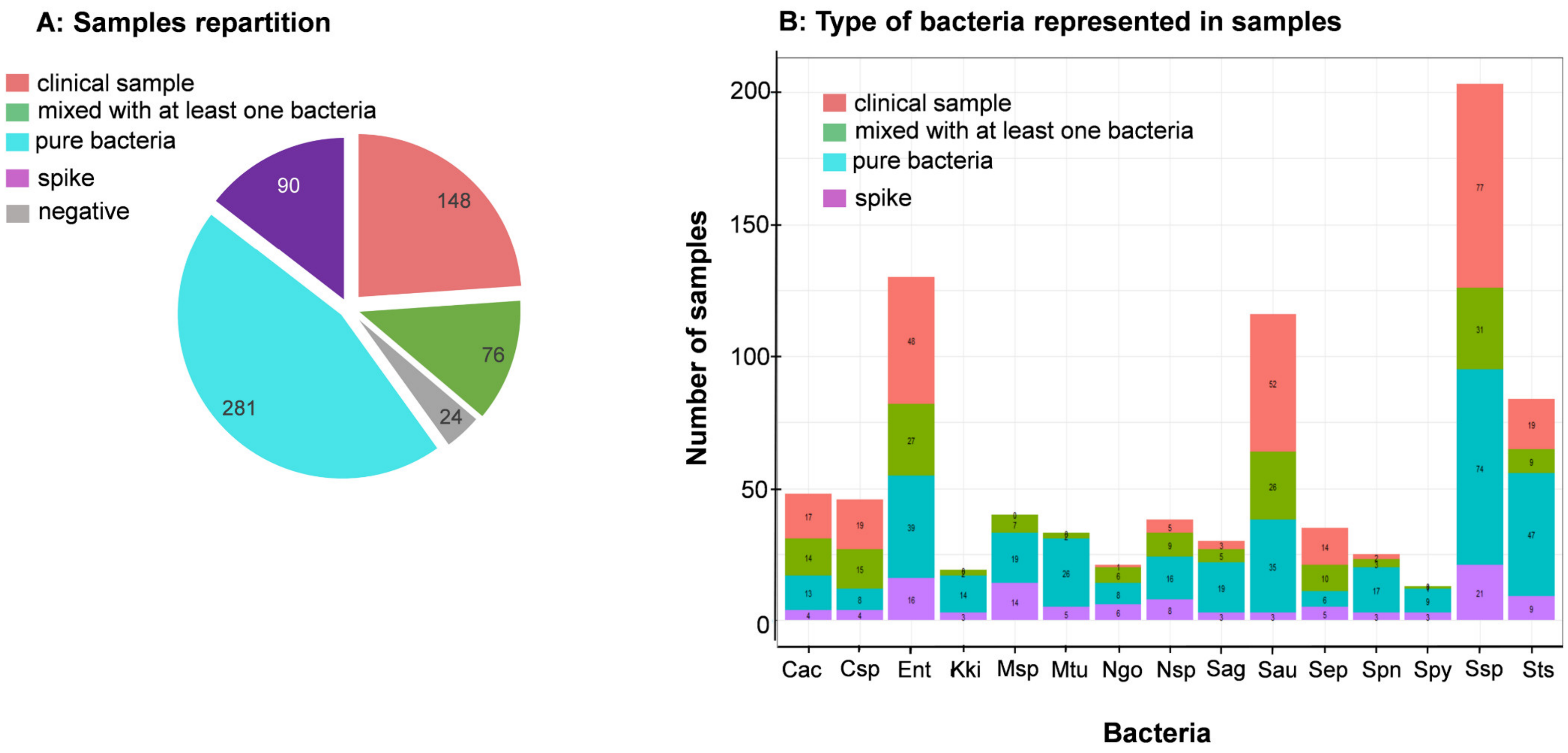

Figure S2. Composition of the training data base. Panel (**A**) shows the samples repartition implemented in the training database. Panel (**B**) show the number and origin of the sample for each specific bacterium that fed the data base. Bacteria are reported by an abbreviated name whose complete name can be found in Table 1 (in the main text).

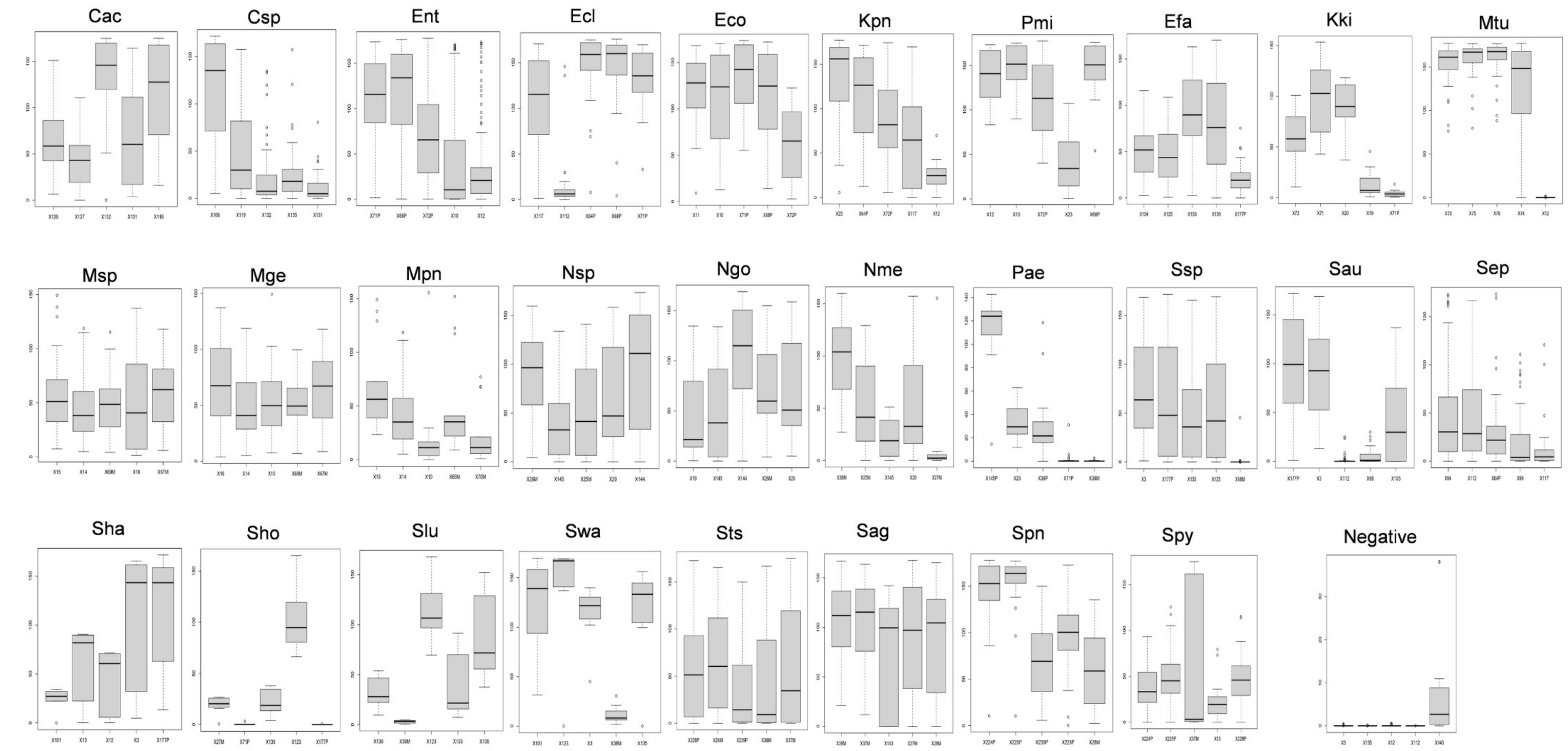

Figure S3. Intensity of oligoprobes designed for each bacterium from the list in Table 1 (main text) on DendrisCHIP®OA expressed as boxplots. The figure shows the oligoprobes enabling the identification of bacteria by our decision algorithm. For the complete name of the bacteria, see Table 1.

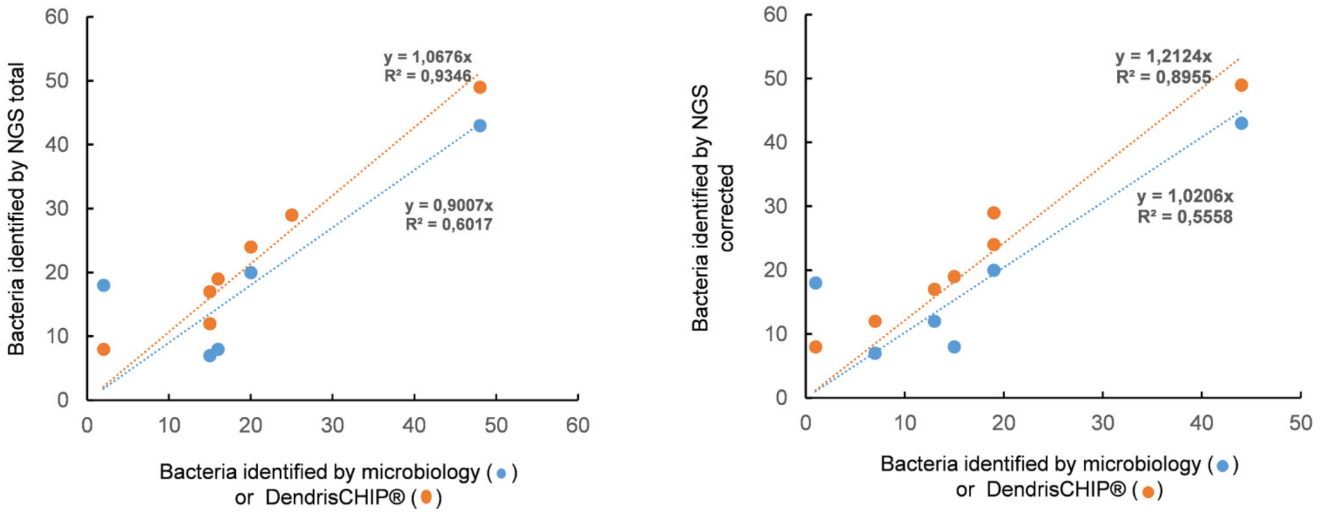

Figure S4. Linear regression analysis of identified bacteria in isolates by DendrisCHIP®OA and microbiology (Microbial cultures) against bacteria identified by NGS as the reference. In left, the linear regression is made with all bacteria identified in the 101 samples and in right, the linear regression is made by excluding bacteria identified only by NGS.

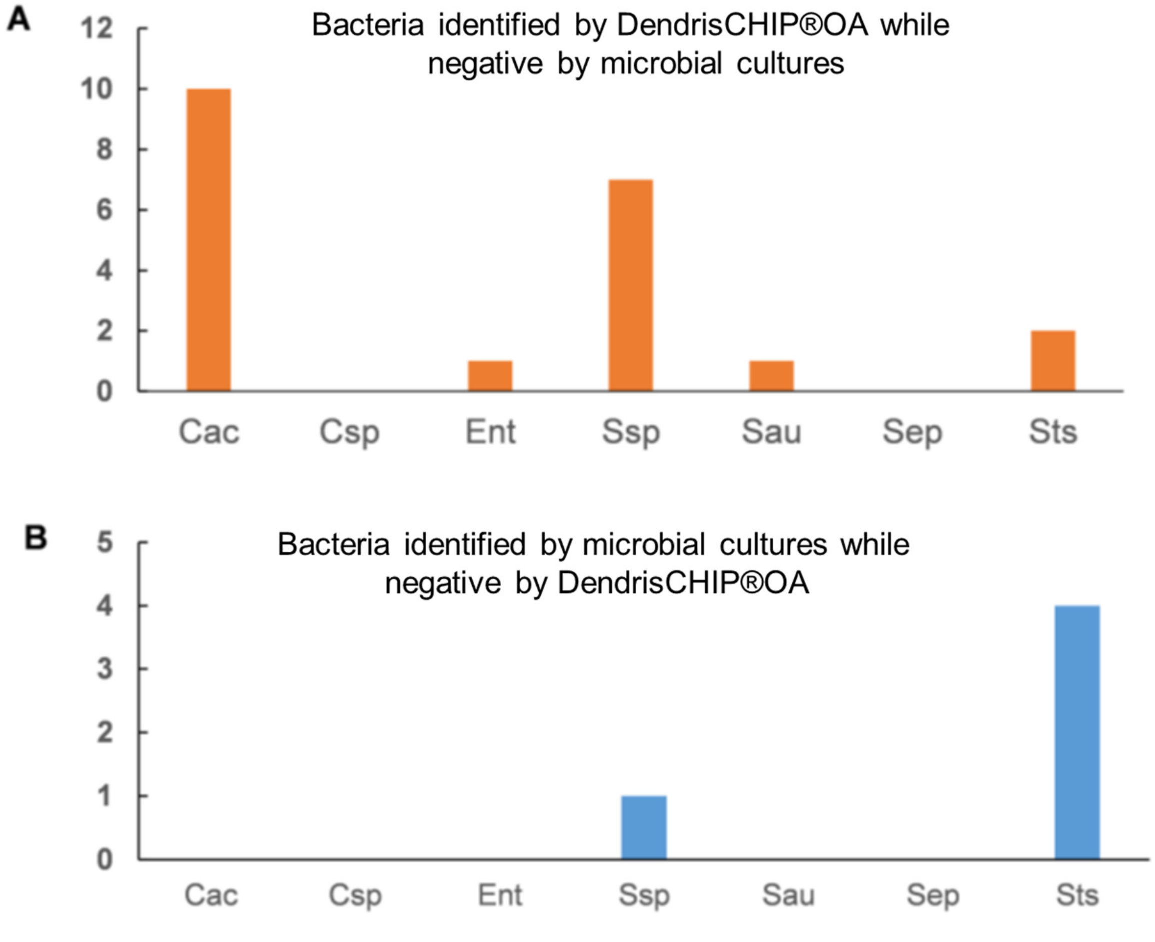

Figure S5. Identification of bacteria in isolates by NGS which are negative for microabial cultures (**A**) ore negative for DendrisCHIP®OA (**B**).

References

32. Untergasser, A.; Nijveen, H.; Rao, X.; Bisseling, T.; Geurts, R.; Leunissen, J.A. Primer3plus, an enhanced web interface to primer3. *Nucleic Acids Res* **2007**, *35*, W71-W74. gkm306 [pii];10.1093/nar/gkm306 [doi].
33. Untergasser, A.; Cutcutache, I.; Koressaar, T.; Ye, J.; Faircloth, B.C.; Remm, M.; Rozen, S.G. Primer3--new capabilities and interfaces. *Nucleic Acids Res* **2012**, *40*, e115. 10.1093/nar/gks596.
13. Fihman, V.; Hannouche, D.; Bousson, V.; Bardin, T.; Liote, F.; Raskine, L.; Riahi, J.; Sanson-Le Pors, M.J.; Bercot, B. Improved diagnosis specificity in bone and joint infections using molecular techniques. *J Infect* **2007**, *55*, 510-517. 10.1016/j.jinf.2007.09.001.
49. Eisenach, K.D.; Cave, M.D.; Bates, J.H.; Crawford, J.T. Polymerase chain reaction amplification of a repetitive DNA sequence specific for mycobacterium tuberculosis. *J Infect Dis* **1990**, *161*, 977-981. 10.1093/infdis/161.5.977.
40. Hwang, S.H.; Kim, D.E.; Sung, H.; Park, B.M.; Cho, M.J.; Yoon, O.J.; Lee, D.H. Simple detection of the is6110 sequence of mycobacterium tuberculosis complex in sputum, based on pcr with graphene oxide. *PLoS One* **2015**, *10*, e0136954. 10.1371/journal.pone.0136954.
50. Martineau, F.; Picard, F.J.; Ke, D.; Paradis, S.; Roy, P.H.; Ouellette, M.; Bergeron, M.G. Development of a pcr assay for identification of staphylococci at genus and species levels. *J Clin Microbiol* **2001**, *39*, 2541-2547. 10.1128/jcm.39.7.2541-2547.2001.
51. Murakami, K.; Minamide, W.; Wada, K.; Nakamura, E.; Teraoka, H.; Watanabe, S. Identification of methicillin-resistant strains of staphylococci by po[lymerase chain reaction. *J Clin Microbiol* **1991**, *29*, 2240-2244. 10.1128/jcm.29.10.2240-2244.1991.
30. Trevisiol, E.; Le Berre-Anton, V.; Leclaire, J.; G, P.; A-M, C.; JP, M.; M, F.J.; B, M. Dendrislides, dendrichips: A simple chemical functionalization of glass slides with phosphorus dendrimers as an effective means for thepreparation of biochips. *New J. Chem* **2003**, *27*, 1713-1719.
